# Spray-Dried Infant Formula Emulsion Stability as Affected by Pre-Heat Treatment and Total Solids Content of the Feed

**DOI:** 10.3390/foods11233752

**Published:** 2022-11-22

**Authors:** Mariana Rodríguez Arzuaga, Analía G. Abraham, Lilia Ahrné, Marvia G. Pérez Montes, María C. Añón

**Affiliations:** 1 Latitud, LATU Foundation, Av. Italia 6201, Montevideo 1500, Uruguay; 2 Centro de Investigación y Desarrollo en Criotecnología de Alimentos (CIDCA), Comisión de Investigaciones Científicas (CIC-PBA) and Consejo Nacional de Investigaciones Científicas y Técnicas (CONICET-CCT La Plata), Universidad Nacional de La Plata (UNLP), CIDCA Calle 47 y 116, La Plata 1900, Argentina; 3 Department of Food Science, Faculty of Science, University of Copenhagen, Rolighedsvej 26, DK-1958 Frederiksberg, Denmark

**Keywords:** denaturation, viscosity, backscattering, milk powder, pasteurization, dry matter

## Abstract

Pre-spray-drying processing may affect stability after reconstitution of emulsion-based powders, such as infant formulas. This study aimed to evaluate the effects of pasteurization temperature and total solids (TS) of the feed on the stability of the emulsions obtained from the reconstituted powders. Four infant formula powders (50%-75 °C, 50%-100 °C, 60%-75 °C, and 60%-100 °C) were produced at pilot scale, from emulsions with 50 or 60% TS pasteurized at 75 or 100 °C for 18 s. Both the emulsion feeds and the emulsions from the reconstituted powders (12.5% TS) were analyzed. The results showed that feeds with 60% TS were flocculated, as indicated by the large particle size and viscosity and the pseudoplastic behavior. Light microscopy revealed that, during spray drying, the flocs were disrupted in 60%-100 °C, while the 60%-75 °C emulsion remained flocculated, reducing its stability post-reconstitution. Although all four emulsions were mainly stabilized by caseins, the presence of β-lactoglobulin was also detected at the oil–water interface, in native state in the formulas preheated at 75 °C and aggregated in the formulas preheated at 100 °C. In conclusion, both the degree of whey protein denaturation (resulting from pasteurization) and the TS of the concentrates during infant formula production affected the emulsion stability of the reconstituted powders.

## 1. Introduction

Global sales of dairy nutritional formulations have been growing in recent years, and the infant formula market in particular is expected to reach USD 53.6 billion by 2027 [1]. Infant milk formulas are oil-in-water emulsions, stabilized by milk proteins and, sometimes, by lecithin or other nonprotein emulsifiers [2].

Infant formulas are often presented in powder form obtained by spray drying. The spray-drying process is widely applied in the dairy industry to produce powders, by the dehydration of a concentrated liquid [3]. The powder characteristics are affected by the composition and rheological properties of the feed and the process parameters (temperatures, air humidity, atomizer type) [4,5,6,7].

Emulsion-based powders, such as infant formulas, should produce stable emulsions after reconstitution and be free of surface-free fat and white flecks, which impair their quality. During infant formula production, heat-induced protein denaturation and dry matter content in the spray dryer feed may affect the emulsion stability of the powdered product.

Infant formulas must be heat-treated during processing to ensure microbial safety. However, subjecting milk-based products to high temperatures may lead to protein denaturation and aggregation, which has technofunctional implications. The interfacial behavior of proteins, acting as emulsifiers in dairy emulsions, varies with their conformational state (native vs. unfolded/aggregated) [8]. Heating emulsions stabilized by whey proteins above their denaturation temperature can promote instability. However, the effect of denaturation on the emulsion stability depends on many factors, such as protein concentration, degree of protein hydrolysis, presence of other proteins (e.g., caseins), and surfactants [9].

Total solids level may also affect the emulsion characteristics. A high dry matter content reduces the distance between particles, increasing the interactions between them [2]. More interactions between particles can lead to more instability, due to flocculation or coalescence mechanisms. On the other hand, in concentrated emulsions, different types of hydrodynamic interactions reduce the creaming velocity of droplets [10].

Increasing the TS of the feed from 50 to 60% and reducing the heat treatment intensity from 100 °C × 18 s to 75 °C × 18 s could be convenient since it significantly reduces the energy consumption during infant formula production [11]. On the other hand, modifying such processing conditions could impair the emulsion stability of the infant formula powders, which is a crucial quality parameter, affecting surface-free fat content, rehydration properties, oxidation, and sticking and caking susceptibility during storage [12]. Although previous studies have focused on the impacts of certain technological and formulation parameters on the emulsion stability of infant formulas [2,4,13,14], the mechanisms through which TS of the feed and pasteurization temperature affect the emulsion stability of infant formula powders after reconstitution have not been reported. Therefore, the objective of this work was to determine the effect of pasteurization temperature (75 and 100 °C) and total solids of the feed (50 or 60%) on the stability of the emulsions obtained by reconstitution of the resulting spray-dried infant formulas.

## 2. Materials and Methods

### 2.1. Materials

Low-heat skim milk powder (SMP), Bützower Dauermilchwerk was purchased from Procudan (Kolding, Denmark). Whey protein isolate (WPI, Lacprodan^®^) and lactose (Variolac 992) were obtained from Arla Foods Ingredients (Viby J, Denmark). Fructo-oligosaccharides (FOS, Beneo Orafiti P95) with a 2–8 degree of polymerization were kindly provided by Alsiano (Birkerød, Denmark). Galacto-oligosaccharides (GOS, Promovita^®^) were kindly provided by Dairy Crest Limited (Edgmond, Newport, United Kingdom). Sunflower oil (SO) was purchased from a local supermarket.

### 2.2. Infant Formula Preparation

Model infant milk formulas (IMFs) were produced in duplicate at the pilot plant of the Department of Food Science of the University of Copenhagen, under four processing conditions: total solids level (TS) of the concentrate = 50 or 60% (*w/w*) and pasteurization temperature = 75 or 100 °C × 18 s. The IMFs were formulated following the European Legislation [15], with a 60:40 whey protein-to-casein ratio, 1:5 total protein-to-lactose ratio, and 1:2.5 total protein-to-oil ratio. 

Each concentrate batch (15 kg) with 50 or 60% TS, was prepared by dispersing the ingredients (SMP, WPI, lactose, FOS, GOS, and SO) in deionized water at 65 °C in a Scanima type SRB-20 mixer (Aalborg, Denmark). Lactose was added to the preheated water and stirred until complete dissolution, followed by approximately 10% of the oil, WPI, SMP, GOS, FOS, and the rest of the oil. All powder ingredients were added under vacuum suction. The pH was adjusted to 6.8 using KOH 2 M, and the mixes were further stirred for 15 min at 65 °C. The concentrates were subsequently pasteurized at 75 °C or 100 °C for 18 s in a MicroThermics Lab tubular heat exchanger (Raleigh, NC, USA) and homogenized using a two-stage APV type B5-15-38 homogenizer (Søborg, Denmark), with a first-stage pressure of 13 MPa, second-stage pressure of 3 MPa, and a flow rate of 2 L/min. The homogenized concentrates were finally spray-dried in a GEA Mobile Minor I spray dryer (Copenhagen, Denmark) equipped with a two-fluid nozzle atomization device. The feed of the spray dryer was kept at 65 °C, and the inlet and outlet air temperatures were 180 and 85 °C, respectively. The average composition of the IMF powders produced under the four processing conditions (50%-75 °C, 50%-100 °C, 60%-75 °C, and 60%-100 °C) was: moisture = 1.1 ± 0.2%, ash = 1.7 ± 0.1%, protein = 11.4 ± 0.2%, fat = 26.7 ± 1.7%, lactose = 55.3 ± 1.9%, GOS = 3.5 ± 0.4% and FOS = 0.4 ± 0.0%.

As determined and reported earlier [11], the pasteurization temperature was the only factor affecting the level of protein of denaturation, which was found to be 8.4 ± 2.7% (*n* = 4) and 82.1 ± 2.3% (*n* = 4) for the IMF powders pasteurized at 75 and 100 °C, respectively. 

### 2.3. Rheological Properties

The rheological properties of the IMF emulsions were measured at the feed of the spray dryer, using an ARES-G2 rheometer (TA Instruments, New Castle, DE, USA), equipped with a concentric cylinder measurement cell. The shear rate was increased from 0 to 300 s^−1^, the temperature was kept at 65 °C, and the shear stress and apparent viscosity data were acquired.

### 2.4. Particle Size Distribution

The particle size distribution (PSD) of the IMF emulsions was determined at the feed of the spray dryer and after spray drying and reconstitution of the powders (12.5% *w/v* in deionized water). The PSD was determined by laser light diffraction using a Mastersizer 3000 (Malvern Instruments Ltd., Worcestershire, UK), with a particle refractive index of 1.46, a dispersant refractive index of 1.33 and a particle absorbance index of 0.001 [16]. The volume mean diameter (D[4,3]) was calculated for each individual population, according to Equation (1).

D[4,3] = ∑n_i_d_i_^4^/∑n_i_d_i_^3^, (1)

where n_i_ is the frequency of appearance of particles in size class i with mean diameter d_i_.

### 2.5. Microstructure

The microstructure of the spray-dried emulsions reconstituted (12.5% *w/v*) in water or SDS 1% (*w/v*) was observed with an Olympus CX21 light microscope (Olympus Corporation of the Americas, PA, USA) using concave slides to avoid droplet deformation. Micrographs were taken with a DinoEye Piece Camera AM4023X (Dino-Lite, CA, USA) at a magnification of 100×.

### 2.6. Protein Composition at the Interface

#### 2.6.1. Powder Reconstitution and Extraction of Adsorbed Proteins

Each spray-dried emulsion was reconstituted in water to a final protein concentration of 1.4% (*w/v*) and stirred at 1500 rpm for 15 s.

The extraction of the proteins adsorbed at the oil–water interface was performed in duplicate for each formula, according to the procedure described by Puppo et al. [17], with some modifications. For the phase separation, 5 mL of a 50% (*w/v*) sucrose solution was mixed with 5 mL of reconstituted emulsion. Then, 4 mL of this mixture was carefully deposited at the bottom of a tube containing 10 mL of a 5% (*w/v*) sucrose solution. The tubes were centrifuged (SIGMA 6-16KS, Sigma Laborzentrifugen GmbH, Osterode am Harz, Germany) for 2 h at 3000× *g* and 10 °C. After centrifugation, two phases were observed: the creamed oil droplets at the top of the tube and the aqueous phase at the bottom. To extract the proteins adsorbed at the interface, 300 µL of the supernatant (cream phase) was homogenized in a vortex with 150 µL of 1% (*w/v*) SDS and centrifuged for 20 min at 10.000× *g* and room temperature in a Spectrafuge 24D centrifuge (Labnet, NJ, USA). The lower phase obtained after centrifugation contained the proteins desorbed from the oil droplets’ surface.

#### 2.6.2. Protein Identification

The protein profiles from the reconstituted emulsions and the oil–water interface (desorbed proteins) were analyzed by SDS-PAGE under reducing and nonreducing conditions. Continuous and stacking gels of 15 and 5% acrylamide, respectively, were prepared. Both the complete reconstituted emulsion and the solution containing the desorbed proteins were mixed with a sample buffer system (0.06 M Tris-HCl buffer pH = 6.8, 25% glycerol, 2% SDS, 0.1% bromophenol blue) with or without 0.72 mM β-mercaptoethanol, in a 4/1 ratio. Mixtures containing β-mercaptoethanol (reducing conditions) were heated in a boiling water bath for 5 min and centrifuged for 5 min at 12.000× *g* and room temperature. Ten microliters of the molecular weight marker (10–180 kDa, #26616, ThermoScientific, USA) and 15 µL of all protein solutions were loaded on the gels. A running buffer system pH = 8.3 containing 0.025 M Tris base, 0.15 M glycine, and 0.8% SDS was used. Electrophoresis was performed using an omniPAGE WAVE Maxi System (Cleaver Scientifics, Rugby, UK) at 200 V. Coomassie brilliant blue was used as a colorant agent. 

### 2.7. Emulsion Stability

Emulsion stability of the spray-dried reconstituted powders was evaluated as a function of time. Each powder sample was reconstituted (12.5% *w/v*) in water and stirred at 1500 rpm for 15 s. Immediately after reconstitution, 5 mL of each emulsion was transferred to a cylindrical glass tube and stored at 20 ± 1 °C for 24 h. The backscattering (BS) of light was measured for each tube at times 0, 2, 3, 5, and 24 h. In addition, the Emulsion Stability Index (ESI) was calculated as reported earlier [11], according to Equation (2).

ESI = (BS_i_[h] − BS_0_[h])/H, (2)

where BS_i_[h] is the BS (%) at time i for position h, BS_0_[h] is the BS (%) at time 0 for position h, and H is the total sample height. 

### 2.8. Statistical Analysis

Two batches of IMF were produced for each processing condition (50%-75 °C, 50%-100 °C, 60%-75 °C, 60%-100 °C) on separate days. Powder reconstitution was performed in duplicate for each batch. Experiments were carried out at least in duplicate for each batch of IMF and/or reconstituted powder. Mean values were subjected to a one-way analysis of variance (ANOVA) and post hoc Tukey’s test, using InfoStat software version 2020 [18]. The level of significance was determined at *p* < 0.05. 

## 3. Results

### 3.1. Rheological Properties

**Figure 1:**
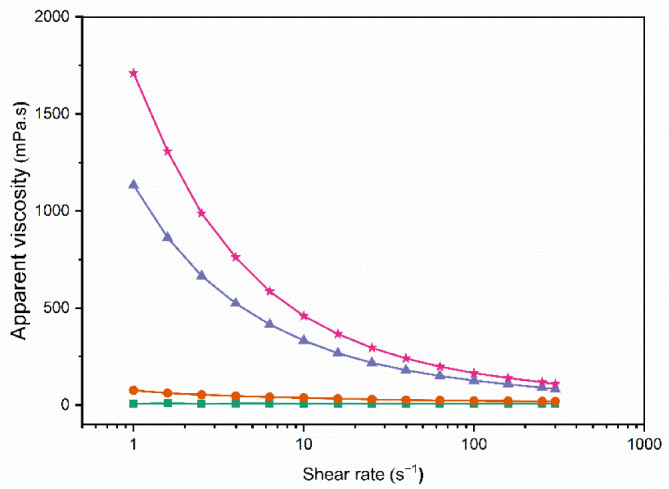
Apparent viscosity of the 50%-75 °C (green square), 50%-100 °C (orange circle), 60%-75 °C (purple triangle), and 60%-100 °C (pink star) IMF feed emulsions versus shear rate (1–300 s^−1^).

**Figure 2:**
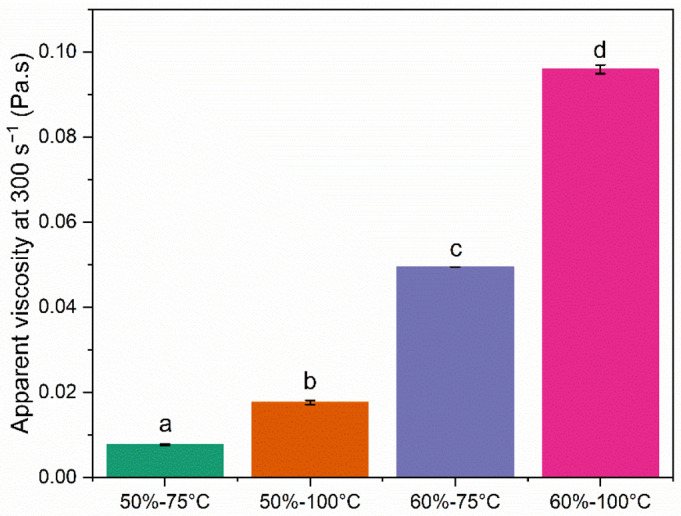
Apparent viscosity of the emulsion feed, determined at 300 s^−1^. Bars indicate the standard deviation (*n* = 4).

The results obtained for the rheological properties of the IMF emulsions, at the feed of the spray dryer, are presented in Figure 1 and Figure 2. The level of TS of the feeds strongly impacted their rheological behavior. While for concentrates with 50% TS, the viscosity was constant with the shear rate, indicating Newtonian behavior, 60% TS concentrates showed pseudoplastic behavior, as the apparent viscosity decreased with the shear rate (Figure 1).

The apparent viscosity of the emulsions (at shear rate = 300 s^−1^) increased significantly (*p* < 0.05) with the TS, as well as with the pasteurization temperature (Figure 2).

### 3.2. Particle Size Distribution

**Figure 3:**
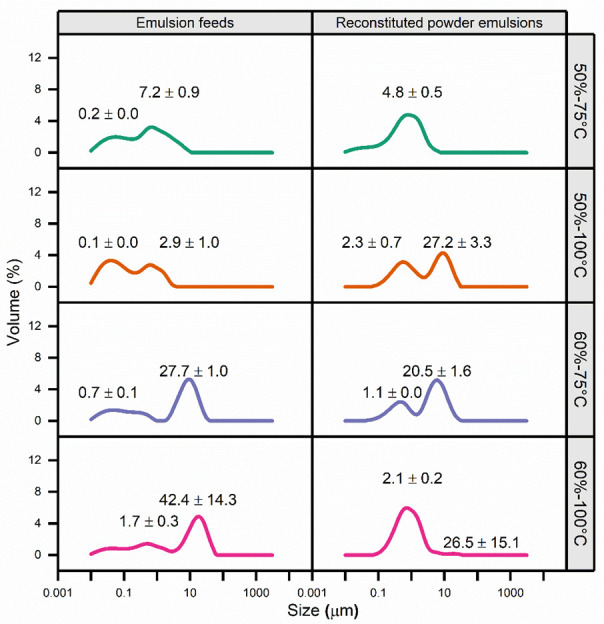
Particle size distribution of the emulsions, before spray drying (feeds) and after spray drying and reconstitution (reconstituted powders). Text on the graphs indicates mean D[4,3] ± standard deviation (n = 4) for each particle population within the sample.

At the entrance of the spray dryer, all four IMF emulsions showed a bimodal PSD (Figure 3). However, while in the 50% TS feeds, the mean particle diameter of the two populations varied approximately between 0.1–0.2 µm and 3–7 µm, in the 60% TS feeds, the mean diameters varied approximately between 0.7–1.7 µm and 27–42 µm. Therefore, the homogenization step was more effective in reducing the particle size of the IMF emulsions with 50% TS. The differences between the particle sizes of the feed emulsions pasteurized at 75 and 100 °C were not significant (*p* > 0.05) for any TS level.

After spray drying and reconstitution, the emulsions preheated at 75 °C (50%-75 °C and 60%-75 °C) showed a slight reduction in the size of the predominant population. On the other hand, the PSD of the emulsions preheated at 100 °C showed important size changes over spray drying and/or reconstitution (Figure 3). The particle size of the 50%-100 °C reconstituted emulsion was significantly larger (*p* < 0.05) than the 50%-100 °C feed emulsion, with the population of larger particles increasing from a mean diameter of 2.9 to 27.2 µm. On the contrary, the particle size of 60%-100 °C was significantly reduced (*p* < 0.05) over the spray-drying and/or reconstitution processes. The mean particle size of the main population in the 60%-100 °C feed emulsion was 42.4 µm, while in the reconstituted emulsion, the main population had a mean particle size of 2.1 µm and a very small population of 26.5 µm.

### 3.3. Microstructure

Since the light scattering method does not allow to differentiate between the growth of individual particles and the association of particles, the reconstituted emulsions were also observed under the light microscope [10].

**Figure 4:**
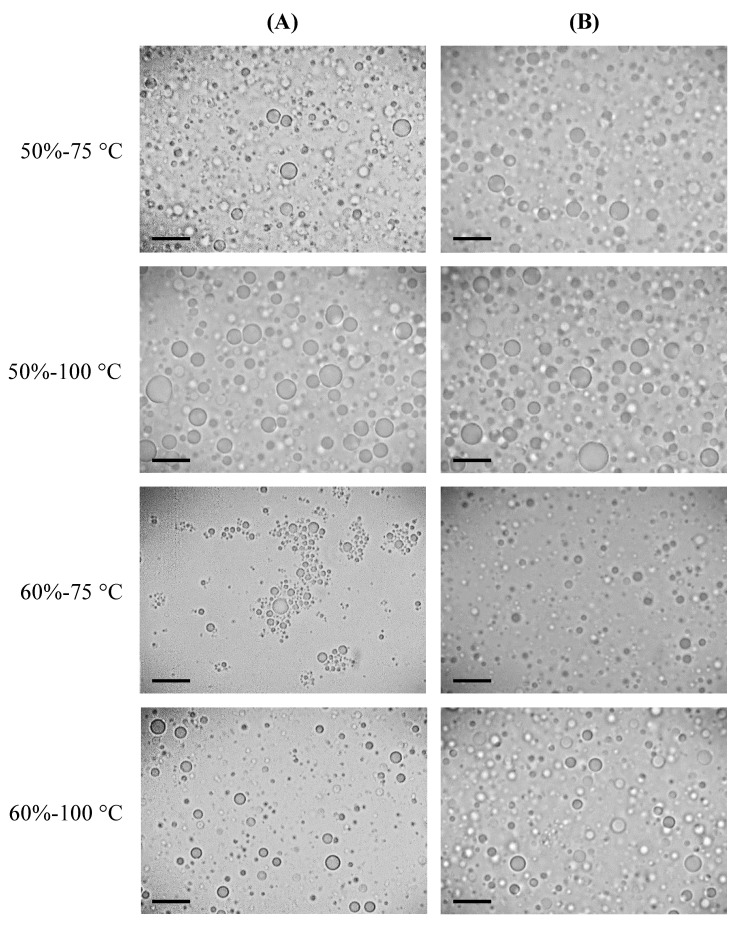
Micrographs (100×) of IMF powder emulsions, reconstituted in water (**A**) and 1% SDS (**B**). Black bars represent 50 µm.

**Figure 5:**
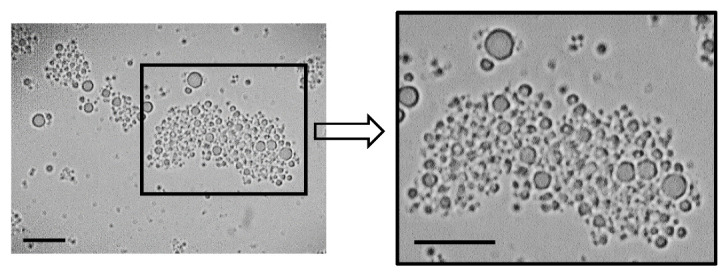
Micrograph (100×) of IMF powder emulsion 60%-75 °C reconstituted in water (**left image**) and magnification of the selected black rectangle (**right image**). Black bars represent 50 µm.

The particle size results obtained by light scattering showed that the 50%-100 °C and 60%-75 °C spray-dried emulsions reconstituted in water presented bimodal distributions, whose main populations had a larger mean diameter than 50%-75 °C and 60%-100 °C (Figure 3). These results were visually confirmed by microscopy (Figure 4A). However, important differences between the 50%-100 °C and 60%-75 °C reconstituted emulsions were evident under the microscope. While 50%-100 °C exhibited droplets of large diameter (Figure 4A), 60%-75 °C was composed of aggregated droplets of smaller size (Figure 4A and Figure 5). The spray-dried reconstituted emulsions were further reconstituted in a 1% SDS solution, to identify the instability mechanism, such as coalescence or flocculation (Figure 4B). SDS is an anionic surfactant that generates strong electrostatic repulsive forces; however, adding SDS did not affect the microstructure of the 50%-100 °C emulsion. Therefore, the increase in particle size observed in 50%-100 °C after spray drying and reconstitution (Figure 3) could be attributed to coalescence. On the other hand, the presence of SDS induced the disaggregation of the oil droplets in 60%-75 °C, indicating that the aggregation mechanism observed in 60%-75 °C reconstituted in water corresponded to flocculation.

An amplified micrograph of 60%-75 °C reconstituted in water (Figure 5) showed that the floc had a compact structure, obtained when the attraction between droplets is relatively weak and retains less continuous phase [10]. This suggests that the aggregation mechanism present in 60%-75 °C was flocculation by depletion, which occurs when there is an excess of protein in the continuous phase [19].

### 3.4. Protein Composition at the Interface

**Figure 6:**
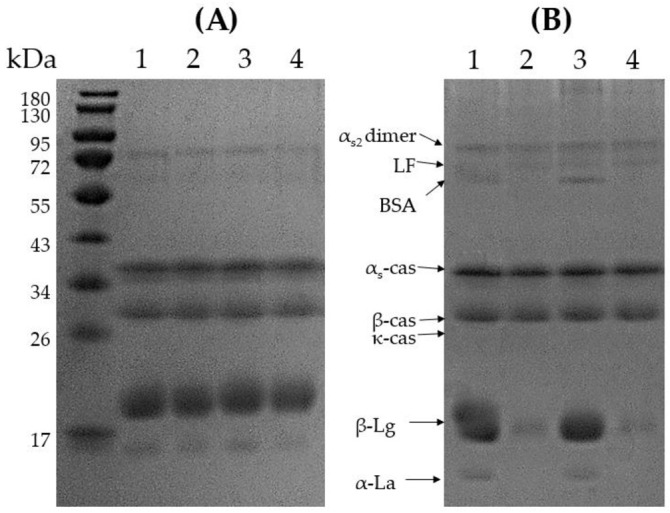
Reducing (**A**) and nonreducing (**B**) SDS-PAGE analysis of the IMF 50%-75 °C (1), 50%-100 °C (2), 60%-75 °C (3), and 60%-100 °C (4). LF = lactoferrin, BSA = bovine serum albumin, cas = casein, β-Lg = β-lactoglobulin, α-La = α-lactalbumin.

Under reducing conditions, no differences were found among the protein profiles of the reconstituted emulsions (Figure 6A, lanes 1–4). The IMFs were formulated to have a 60:40 whey protein to casein ratio, and, considering that β-lactoglobulin (β-Lg) comprises ~50% of the total whey proteins [20], it represents ~30% of the total protein present in the IMFs. Under nonreducing conditions (Figure 6B), in 50%-100 °C (Lane 2) and 60%-100 °C (Lane 4), α-lactalbumin (α-La) and bovine serum albumin (BSA) were almost imperceptible, while β-Lg was significantly reduced. These changes were not observed for 50%-75 °C and 60%-75 °C, indicating that pasteurization at 100 °C during IMF production provoked protein aggregation through reducing bonds, such as disulfide bridges, inducing the formation of high-molecular-weight aggregates that did not enter the gel, and was, therefore, not detected under nonreducing conditions (Figure 6B).

**Figure 7:**
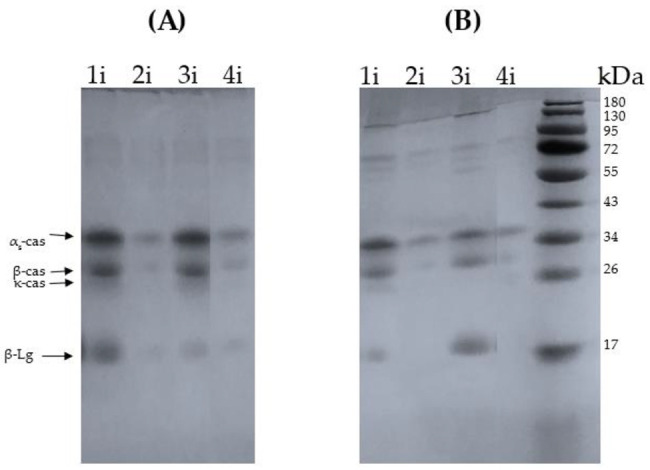
Reducing (**A**) and nonreducing (**B**) SDS-PAGE analysis of the proteins adsorbed at the oil–water interfaces from the 50%-75 °C (1i), 50%-100 °C (2i), 60%-75 °C (3i), and 60%-100 °C (4i) IMFs. β-Lg = β-lactoglobulin, cas = casein.

Although the IMFs contained a higher proportion of WP, caseins predominated at the oil–water interface of all four emulsions (Figure 7). The preferential adsorption of caseins over whey proteins in dairy systems has been reported and explained by their higher flexibility and hydrophobicity [2,21,22,23,24]. α-La was not detected at the interface, while β-Lg was only detected at the interfaces of the emulsions pasteurized at 75 °C (1i and 3i). The IMFs pasteurized at 100 °C presented the lowest levels of proteins (caseins and β-Lg) adsorbed at the emulsion interface (lanes 2i and 4i). The lower protein content at the interface of 50%-100 °C and 60%-100 °C could be explained by the whey protein denaturation and whey protein–whey protein and whey protein–casein aggregation, which affects the flexibility of the proteins impairing their ability to locate at the interface [25,26].

IMFs heat treated at 75 °C presented caseins and β-Lg at the oil–water interface, which was evidenced when analyzing the protein profiles both under reducing and nonreducing conditions (Figure 7A,B). On the other hand, the emulsions pasteurized at 100 °C, where whey protein denaturation had already occurred, presented only casein at the interface (Figure 7A,B). The analysis of the total protein profiles revealed that the pasteurization at 100 °C induced the aggregation between whey proteins and caseins mediated by disulfide bonds (Figure 6A,B, Lanes 2 and 4). According to the obtained results in Figure 6A and B such aggregates remained in the bulk solution and were not incorporated in the interface.

### 3.5. Emulsion Stability

**Figure 8:**
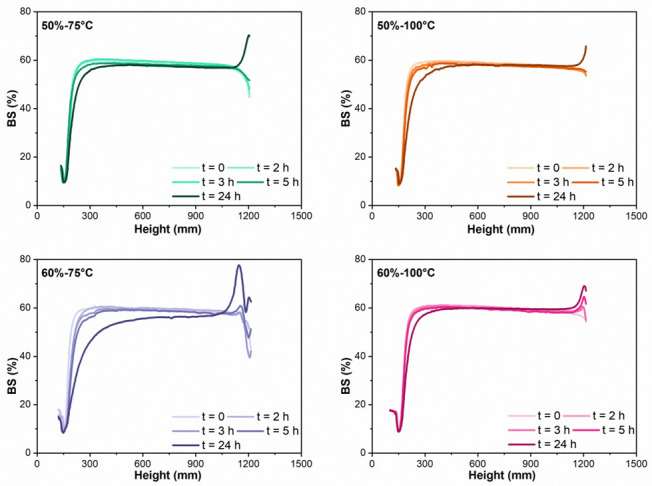
Backscattering (BS,%) profiles of the reconstituted IMF emulsions, after 0, 2, 3, 5, and 24 h of storage at 20 °C.

An increase in backscattering (BS) at the top part of the tube and a reduction in the lower part, indicate creaming and/or flocculation. Such behavior was observed in the four reconstituted spray-dried emulsions, after 24 h of storage (Figure 8).

**Figure 9:**
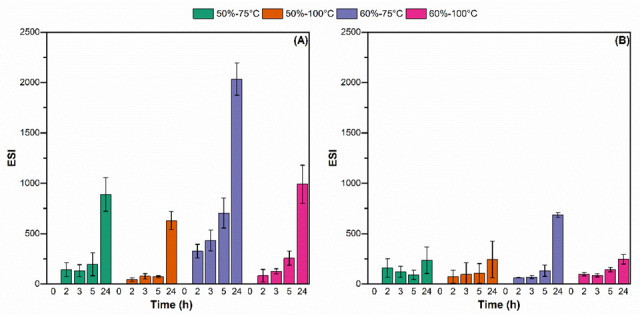
Emulsion stability index (ESI) at the lower ((**A**), height = 2–3 cm) and higher ((**B**), height = 10–12 cm) of the reconstituted 50%-75 °C (green), 50%-100 °C (orange), 60%-75 °C (purple), and 60%-100 °C (pink) IMF emulsions, after 0, 2, 3, 5, and 24 h.

A deeper analysis of the emulsion destabilization was carried out through the calculation of an emulsion stability index (ESI, Figure 9). A higher ESI value indicates more destabilization (Equation (2)). In emulsions obtained from feeds with 50% TS (50%-75 °C and 50%-100 °C) a significant destabilization (*p* < 0.05) at the lower part of the tube was only detected at the last measurement (24 h of storage) (Figure 9A), while no significant changes (*p* > 0.05) were obtained at the top part of the tube, even one day after reconstitution (Figure 9B). A significant increase in ESI was obtained after 5 h at the lower part and after 24 h at the top part of the 60%-100 °C emulsion. Finally, 60%-75 °C was the first emulsion to show destabilization signs, after 2 h in the lower part and 5 h in the upper part (Figure 9).

## 4. Discussion

During the production of the formulas, the emulsions undergo changes that may depend on the processing parameters. First, the ingredients were dispersed, and the proteins located at the oil–water interface formed the emulsion. Then, the concentrates were heat treated, which may enhance or reduce the emulsions’ stability, mainly due to protein denaturation and aggregation, depending on the pasteurization temperature. The emulsions were subsequently homogenized, before entering the spray dryer, with the aim of improving the stability of the emulsions by reducing the oil droplet size. Both the TS of the emulsions before spray drying (50 or 60%) and the pasteurization temperature (75 or 100 °C) affected the characteristics of the spray-dried emulsions.

Before spray drying, the 50% TS feed emulsions presented the smallest particle size (Figure 3), Newtonian behavior (Figure 2), and the lowest viscosity (Figure 1). Emulsion 50%-75 °C presented a significantly lower (*p* < 0.05) viscosity than 50%-100 °C, probably explained by the higher degree of denaturation in the latter. When denaturation of whey proteins occurs, the voluminosity of whey proteins increases, and the effective volume of the casein micelles increases as well as a result of the whey protein–casein interactions and the association between casein micelles [27].

The 60% TS feed emulsions presented larger particle sizes than the 50% TS feed emulsions (Figure 3). At higher TS levels, the intermolecular distances are reduced and the interactions between proteins adsorbed and nonadsorbed at the interface are favored. Such interactions may lead to the formation of flocs of oil droplets and a subsequent increase in viscosity [28], as was observed in Figure 2. In addition to the higher viscosity, emulsions with 60% TS showed pseudoplastic behavior (Figure 1). These results suggest that the homogenization process was not completely effective for the 60% TS emulsions. The two-stage homogenization process aims to reduce the particle size of the droplets, at the first stage carried out at high pressure (13 MPa), and to disaggregate the flocs that may have been formed after the first stage, at the second stage carried out at a lower pressure (3 MPa) [10,29]. Based on the results obtained for the 60% TS emulsions, it can be hypothesized that the first stage of homogenization reduced the size of the individual oil droplets, while the second stage did not achieve the disruption of the flocs formed. Indeed, the presence of flocs explains the large particle size obtained for the 60% TS emulsions after homogenization (>20 µm, Figure 3), the higher viscosities (Figure 1), and the pseudoplastic behavior (Figure 2). In flocculated emulsions, the flocs are deformed and disrupted as the shear rate increases, leading to a decrease in viscosity, which explains the pseudoplastic behavior [30].

After spray drying and reconstitution of the powders, the 50%-75 °C emulsion presented a slight reduction in the particle size, while a bimodal PSD was obtained for 50%-100 °C with a population of significantly larger particle size (~27 µm, Figure 3), indicating that flocculation or coalescence occurred during reconstitution. Similar results were reported by Drapala et al. [4] for infant milk formula powders after reconstitution. The comparison of the micrographs obtained for the 50%-100 °C powder reconstituted in water and SDS revealed that the oil droplet aggregation mechanism present was coalescence (Figure 4). Dapueto et al. [31] observed an increase in the oil droplet diameter with the degree of protein denaturation. The authors attributed the increase in the particle size to the reduction of native whey proteins, which decreases their ability to stabilize the new interfaces created during the homogenization process. In our study, the spray dryer nozzle may have disrupted the oil droplets, and, while for 50%-75 °C it implicated a slight particle reduction, the new droplets created in 50%-100 °C coalesced during reconstitution. As shown by SDS-PAGE, whey proteins in 50%-100 °C were extensively denatured and aggregated with the caseins (Figure 6), which diminished their presence at the oil–water interface (Figure 7).

The particle size reduction obtained after spray drying for 60%-100 °C also indicates that the shear applied by the nozzle during spray drying disrupted the flocs formed after the homogenization step, as explained above. This result was confirmed by light microscopy, where 60%-100 °C showed nonaggregated oil droplets (Figure 4). On the other hand, 60%-75 °C was still flocculated after spray drying and reconstitution (Figure 4 and Figure 5). The different behavior obtained for 60%-75 °C and 60%-100 °C during spray drying and/or reconstitution may be explained by the presence of flocs of larger size and denatured/aggregated proteins in the bulk of the 60%-100 °C emulsion, which generated interactions susceptible to destabilization during spray drying.

Overall, all four emulsions presented different characteristics after reconstitution, which can be attributed to their different processing conditions (pasteurization temperature and TS in the concentrate). In the case of the 50%-75 °C emulsion, the proteins were in native state, and both caseins and β-Lg could locate at the interface, where there was enough protein content to form a viscoelastic film, ensuring a stable emulsion. When the TS level of the concentrate was increased to 60% (emulsion 60%-75 °C), there was a higher content of protein at the bulk phase, which may favor the flocculation by depletion observed in that sample, through interactions between caseins and β-Lg adsorbed and nonadsorbed at the interface. In 50%-100 °C, the whey proteins were denatured, and whey protein–casein and whey protein–whey protein aggregates were formed. The 50%-100 °C emulsion was only stabilized by caseins, probably because the aggregated β-Lgs lost their ability to interact with the interface. The lower TS level (50%) and the smaller amount of protein at the interface resulted in a thinner viscoelastic film that favored coalescence during reconstitution. By contrast, in 60%-100 °C, the higher TS level allowed the formation of a thicker viscoelastic film or the increase in the viscosity of the bulk phase, preventing the coalescence. Further, the higher degree of denaturation and aggregation in 60%-100 °C compared to 60%-75 °C may have contributed to form weaker flocs after homogenization, which were disrupted over spray drying.

The flocculation of the 60%-75 °C emulsion led to reduced stability after reconstitution (Figure 9). In diluted emulsions, such as the reconstituted infant formulas (12.5% TS), flocculation favors creaming because the effective size of the particle increases [10]. On the other hand, 50%-100 °C, which presented a large particle size due to coalescence during reconstitution, showed good stability. The difference between these two samples can be explained by the fact that, while the large particle size observed in 60%-75 °C corresponded to aggregated droplets, in the case of 50%-100 °C, there were individual droplets of large diameter, as confirmed by microscopy. In the case of 60%-75 °C, the flocs rise with storage time and grow, which in turn accelerates the migration to the surface, forming the upper cream layer.

## 5. Conclusions

Both the degree of protein denaturation and/or aggregation, induced by heat treatment, and the TS level of the emulsions affected their behavior during and after processing. The 50% TS emulsion feeds showed low viscosity and Newtonian flow behavior after homogenization, while homogenization was not effective in 60% TS emulsions, resulting in flocculation, high viscosity, and pseudoplastic behavior.

Spray drying and reconstitution of the formulas pasteurized at 75 °C did not have a significant impact on the characteristics of the emulsions. Moreover, 60%-75 °C was still flocculated after spray drying and was the first formula in showing destabilization signs after reconstitution.

In the case of the formulas pasteurized at 100 °C, whose whey proteins were extensively denatured, important changes occurred during spray drying and reconstitution. Spray drying disrupted the flocs in 60%-100 °C, which showed no sign of droplet aggregation after reconstitution. Coalescence occurred during spray drying and/or reconstitution of 50%-100 °C powder emulsion, as indicated by an increase in the individual oil droplet size. However, the increase in the particle size did not reduce the emulsion stability.

The emulsions with a high degree of protein denaturation and aggregation (pasteurized at 100 °C) were stabilized by caseins, and their presence at the oil–water interface was reduced, due to interactions with the denatured whey proteins present in the continuous phase. The emulsions pasteurized at 75 °C, which contained native whey proteins, were also mainly stabilized by caseins, although β-Lg was also present at the interface. Further, 60%-75 °C showed a larger amount of native β-Lg than 50%-75 °C, which may explain its tendency to flocculate by depletion.

## Data Availability

Data is contained within the article.
